# Inter-session variability of muscle synergies during upper limb elevation movements in healthy subjects

**DOI:** 10.1007/s00221-026-07297-8

**Published:** 2026-04-19

**Authors:** Valentina Lanzani, Francesco Scandelli, Federico Temporiti, Francesca Cappelletti, Luca Canova, Paola Adamo, Roberto Gatti, Alessandro Scano

**Affiliations:** 1 Advanced Methods for Biomedical Signal and Image Processing Laboratory, Institute of Intelligent Industrial Systems and Technologies for Advanced Manufacturing (STIIMA), Italian Council of National Research (CNR), Milan, Italy; 2 Physiotherapy Unit, IRCCS Humanitas Research Hospital, Rozzano, Milan, Italy; 3 Department of Biomedical Sciences, Humanitas University, via Rita Levi Montalcini 4, 20072 Pieve Emanuele, Milan, Italy; 4 Humanitas University, via Rita Levi Montalcini 4, 20072 Pieve Emanuele, Milan, Italy

**Keywords:** Muscle synergies, Inter-session variability, Surface electromyography, Upper limb movements

## Abstract

Muscle synergies provide a framework to study motor control in healthy and clinical populations through electromyographic recordings during motor tasks. However, their variability has been poorly investigated. This study aimed to assess inter-session variability of muscle synergies during elevation tasks performed with the dominant and non-dominant upper limbs in healthy subjects. Fifteen young healthy adults performed explosive arm elevation movements along the sagittal, scapular and adduction planes, starting with the arm alongside the body and reaching a 90° angle relative to the trunk. Electromyographic signals were recorded from 20 muscles (agonist and postural muscles of upper limbs, trunk and lower limbs). Data were collected at baseline (T0) and after 1 day (T1) without any intervention. Four synergies (Upper limb acceleration synergy, Upper limb deceleration synergy, Postural adjustment synergy 1 and Postural adjustment synergy 2) were extracted per session using non-negative matrix factorization. Cosine similarity and non-parametric statistics were used to assess inter-session consistency. Results showed significant inter-session similarity of muscle synergies (0.86 for the dominant limb, 0.78 for the non-dominant limb), compared to random similarity (0.48 and 0.47, respectively). The dominant arm exhibited higher reproducibility than the non-dominant arm (*p* = 0.002). Upper limb acceleration synergies were the most stable, whereas Postural adjustment synergies (1 and 2) showed greater variability, especially in the non-dominant arm. Muscle synergies during upper limb elevation exhibit moderate-to-high repeatability in healthy subjects, but non-negligible variability was found, reflecting physiological differences and potential methodological factors. Quantifying this variability may support interpretation of motor control changes in longitudinal studies.

**Supplementary Information:**

The online version contains supplementary material available at 10.1007/s00221-026-07297-8.

## Introduction

Surface electromyography (s-EMG) offers valuable insights for studying motor control in humans (Liu et al. 2024). The central nervous system’s ability to coordinate and control multiple muscles during voluntary movements remains a longstanding question (D’Avella and Bizzi 2005). A widely accepted theory suggests that the central nervous system simplifies the control of complex motor tasks through a coordinated activation of groups of muscles over time, known as muscle synergies (Bizzi et al. 2008). Muscle synergies are usually extracted from EMG signals using matrix factorization algorithms, such as non-negative matrix factorization (NMF). This method identifies recurrent patterns of muscle activation and represents each synergy as a set of weights linearly combined with temporal activation profiles that quantify the contribution of each muscle included in the synergy (Tresch et al. 2006).

Muscle synergies have been adopted to investigate motor control in healthy individuals and subjects with clinical conditions (B. R. Shuman et al. 2017) in various fields, such as gait analysis, sports, robotics and neurorehabilitation (D’Avella et al. 2006; Zhao et al. 2023). In particular, muscle synergies have been used as a neurophysiological marker to quantify the impairment severity and effects of therapy or training during functional tasks (Jobson et al. 2013; Shuman et al. 2016). In this context, muscle synergies analysis could provide valuable support for diagnosis and treatment planning. However, the variability of muscle synergies needs to be quantified in order to adopt muscle synergy as an assessment tool, especially in longitudinal study designs (Shuman et al. 2016). Variability is typically assessed using similarity-based metrics comparing muscle synergies extracted from data collected on the same subjects during multiple experimental sessions, in order to quantify inter-session variability. Specifically, inter-session variability analysis consists of comparing muscle synergies across time-points, considering the intrinsic physiological changes of motor performance (e.g., adaptation, fatigue, etc.) and extrinsic factors including potential differences in electrodes placement and signals collection (Pale et al. 2020). Indeed, subjects may execute the same movement with slightly different neuromuscular activity and kinematics, leading to the extraction of muscle synergies that are not identical between sessions, even in healthy subjects. Such a variability may derive from physiological fluctuations in task performance or as a result of daily-dependent changes in electromechanical conditions of soft tissues (Liu et al. 2024). Additionally, technical factors such as electrode placement, signal noise, and skin impedance may introduce variations in EMG signals and affect muscle synergies similarity across sessions, being NMF sensitive to the quality and consistency of input data. Moreover, EMG pre-processing pipelines including high-pass filtering, rectification, low-pass filtering, and normalization may also influence the final output. In fact, small raw signal fluctuations may be amplified or attenuated during pre-processing, altering the data structure provided to the NMF algorithm (Kieliba et al. 2018).

The extent to which these sources of variability affect muscle synergies extraction is crucial for ensuring reliable assessments, understanding whether changes in synergy patterns following a therapy or training program reflect the physiological variability, methodological artifacts or actual changes in motor performance (Pale et al. 2020). Therefore, inter-session variability assessment is clinically relevant for diagnosis and treatment planning, but its investigation represents the first mandatory step to accurately interpret longitudinal modifications of muscle synergies (Shuman et al. 2016). To date, a few studies have investigated muscle synergies variability during a longitudinal assessment(Allen et al. 2017; Kristiansen et al. 2016) or multi-session study design (Pale et al. 2020), however, the latter have mainly focused on intra- or inter-subject comparisons or longitudinal analyses of muscle synergies after training or therapy (Allen et al. 2017; Rimini et al. 2017; Scano et al. 2019). In contrast, very few studies have examined muscle synergies similarity in healthy subjects performing movements across sessions under identical biomechanical conditions. Moreover, such analysis was limited to the distal part of the upper limb (e.g., hand grasps) (Pale et al. 2020). In this scenario, longitudinal studies are needed to assess the variability of muscle synergies during a motor task performed across multiple sessions in the absence of any intervention or training in between.

Therefore, the aim of this study was to investigate the inter-session variability of muscle synergies in healthy subjects who performed standardized motor tasks in two different days. The tasks consisted of a multi-planar explosive arm elevation movement at 90°. During such tasks, we investigated whether muscle synergies remained stable over time in young healthy subjects who are familiar with such movement. The experimental setting included a bilateral total-body protocol that considered agonist muscles responsible for the movement execution and postural muscles contributing to postural adjustments and intra-segmental fixation strategies.

## Methods

### Participants and setting

Fifteen young healthy subjects (8 males, mean age 22.88 ± 1.81 years, mean height 178.25 ± 5.34 cm, mean weight 74.44 ± 11.86 kg; 7 females, mean age 21 ± 0.58 years, mean height 169.71 ± 9.32 cm, mean weight 61 ± 11.4 kg) were enrolled in this study, which took place in two separate sessions at one day distance. All participants were healthy right-handed subjects without motor, functional and neurological impairments. Written informed consent was obtained by each subject before inclusion in the study. The study was conducted at the Motion Analysis Laboratory of IRCCS Humanitas Research Hospital of Rozzano, Milan, Italy. The study protocol (n. CLF24/01) was approved by the Lombardy Territorial Ethical Committee 5 (CET5) on the 27th August 2024 and conducted in compliance with the Declaration of Helsinki.

### Experimental set-up

**Fig. 1:**
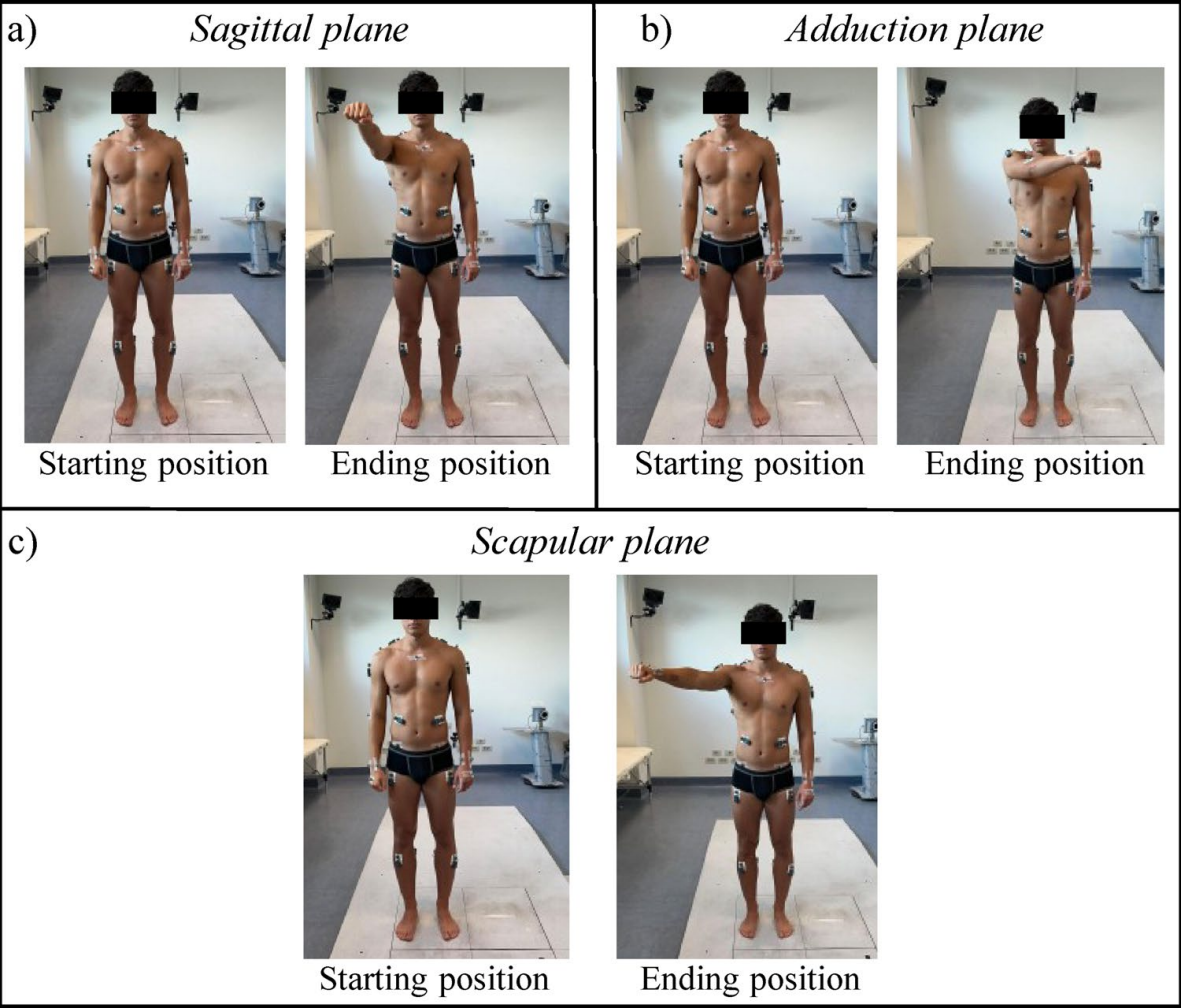
Arm elevation movements performed by the participants during 2 sessions with both upper limbs.** a** Subject starts with the arm resting alongside, then elevates the arm at 90° along the sagittal plane.** b** Subject starts with the arm resting alongside, then elevates the arm at 90° along the adduction plane.** c** Subject starts with the arm resting alongside, then elevates the arm at 90° along the scapular plane

Participants were asked to maintain an upright posture at the center of the Motion Analysis Laboratory, inside the acquisition volume defined by an 8-camera optoelectronic motion capture system (SMART-DX, BTS Bioengineering, Milan, Italy). Participants were instructed to maintain their arms alongside and subsequently to perform explosive arm-raising movements along the required plane (sagittal, scapular, or adduction plane) until reaching approximately shoulder height (about 90° relative to the trunk) (Fig. 1). No real-time kinematic feedback was provided. Each time the arm was elevated, it was held at the final position for 2 s before returning to the starting posture. This movement was performed 6 times along each plane and performed bilaterally. A 2-second pause interspaced each repetition, and a 5-minute of rest was provided before changing the movement plane, in order to minimize fatigue. Prior to data collection, subjects performed familiarization trials. All movements execution was supervised by the operator to ensure consistency with the task instructions.

The dimension of the base of support was standardized and maintained constant across testing sessions (T0 and T1) of each participant. To standardize foot placement, feet were maintained parallel, and the distance between heels was measured at the beginning of each recording session to replicate the same base of support during all tasks and sessions. The order of the movement planes and the starting arm were randomized for each participant.

In each session, 16 hemispherical retro-reflective markers (6 mm diameter) were placed on anatomical landmarks of upper limbs and trunk for the collection of kinematic data. In particular, 8 markers were used to identify the trunk, two placed on the jugular incisura and the seventh cervical vertebra, two on the acromial angle of each shoulder, two bilaterally on the anterior superior iliac spines (ASIS), one on the sacrum. Additionally, 8 markers were bilaterally positioned on the lateral epicondyle of the humerus, on the radial and ulnar styloid processes and between second and third metacarpal bones of the hands. Kinematics data were collected at 100 Hz using 8-camera optoelectronic motion capture system (SMART-DX, BTS Bioengineering, Milan, Italy).

**Fig. 2:**
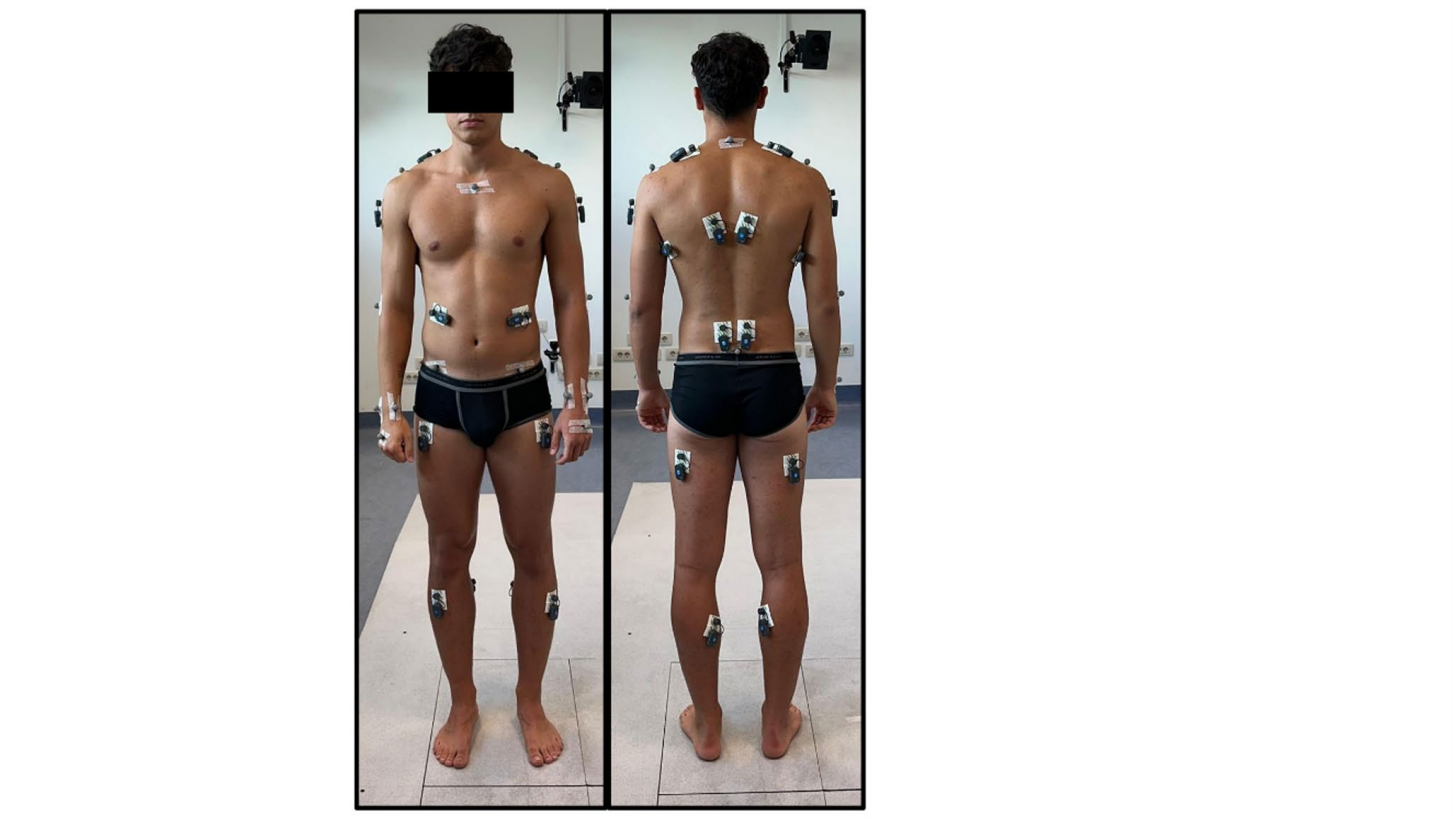
Participants wore a set of 16 hemispherical retro-reflective markers and 20 s-EMG electrodes. Markers were placed bilaterally on the trunk and upper limbs: on the jugular incisura, seventh cervical vertebra, acromial angle of the shoulder, anterior superior iliac spines, lateral epicondyle of the humerus, radial and ulnar styloid processes and between second and third metacarpal bones of the hand. Muscles of both sides of the body were included: Middle Deltoid (MD), Upper Trapezius (UT), Lower Trapezius (LT), Erector Spinae Longissimus (ERL), Latissimus Dorsi (LD), Obliquus Externus Abdominis (OEA), Biceps Femoris Long Head (BF), Rectus Femoris (RF), Gastrocnemius medialis (GM), Tibialis Anterior (TA)

Surface EMG signals were acquired at a sampling rate of 1000 Hz using wireless probes (BTS FREEMG-1000, Milan, Italy), equipped with Ag/AgCl bipolar surface electrodes (FIAB Spa, Florence, Italy). The inter-electrode distance was 20 mm. 20 s-EMG electrodes were placed on the following muscles bilaterally: Middle Deltoid (MD), Upper Trapezius (UT), Lower Trapezius (LT), Erector Spinae Longissimus (ERL), Latissimus Dorsi (LD), Obliquus Externus Abdominis (OEA), Biceps Femoris Long Head (BF), Rectus Femoris (RF), Gastrocnemius medialis (GM), Tibialis Anterior (TA) (Fig. 2). These muscles were selected to allow for the analysis of agonist and postural muscles in a movement where they are recruited for different functions during tasks. Electrode placement followed SENIAM guidelines (Hermens et al. 2000) when available; for muscles not included in SENIAM recommendations, electrodes were positioned over the medial belly of the muscle and aligned with the direction of muscle fibers. Prior to electrode placement, the skin was prepared by shaving the area when necessary and cleaning with alcohol to reduce skin impedance. EMG and kinematic data were synchronized at hardware level by the acquisition system, Smart Capture software (BTS Bioengineering, Milan, Italy).

The whole protocol had a duration of approximately 1 h per subject. Each subject participated in two experimental sessions (T0 and T1) at 1 day distance, where the recording session was repeated by replacing markers and electrodes.

### Data analysis and synergy extraction

The data collected in the two sessions (T0 and T1) were processed independently, following the same analysis pipeline. The first step of data analysis consisted of a preliminary processing of all kinematics data using the Smart Tracker software (BTS Bioengineering, Milan, Italy). This software allows for reconstruction of the three-dimensional position of each marker based on the frames collected via the optoelectronic system. The second step consisted of data processing using MATLAB 2023b (Mathworks, Natick, Massachusetts, United States), with a custom-developed software. During data elaboration, EMG data were high-pass filtered at 30 Hz (Butterworth filter, 6th order) to remove motion artifacts, rectified, and low-pass filtered with a cut-off frequency of 8 Hz (Butterworth filter, 6th order) to extract the EMG envelope. EMG envelopes obtained from neuromuscular activity of all recorded muscles (upper-limbs, trunk and lower limbs) involved during movements along three planes of motion were concatenated into a single matrix for each subject. Subsequently, EMG envelopes of each muscle were normalized to its maximum activation value recorded across all trials within the same session (T0 or T1). In this way, the EMG waveforms were scaled into an interval of values between 0 and 1, achieving normalized EMG envelopes. Then, the specific-session normalized EMG signal was segmented according to the arm motion along the three planes, considering a single arm per time.

To separate the movements into distinct phases, kinematic data were analyzed. Although a full marker set was recorded, the present study focused on inter-session variability of muscle synergies rather than on biomechanical characterization of the movement. Therefore, kinematic data were primarily used to identify movement onset and offset for EMG segmentation, and no additional kinematic variables were further analyzed. Indeed, the only kinematic information used to identify six repetitions per plane was the velocity profile of the elbow marker. The elbow marker trajectory was low-pass filtered at 5 Hz (Butterworth filter, 7th order), and its first derivative was computed to obtain the velocity signal. Individual movements were identified by detecting velocity peaks exceeding a predefined threshold (30% of the maximum velocity amplitude). Zero-crossing points were computed as sign changes between consecutive samples of the velocity signal. For each velocity peak, movement onset was defined as the zero-crossing immediately preceding the peak, while movement offset was defined as the subsequent zero-crossing, thereby defining the temporal boundaries of each movement. To enable data comparison, all movements were time-aligned by extracting EMG signals within the interval [− 0,5; + 1,0] seconds relative to the movement kinematic onset (aligned at 0 s for all movements). This procedure allowed us to capture the complete EMG waveforms which began before movement kinematic onset and finished after having reached the target. To concatenate the EMG envelopes from the three movement planes into a single matrix suitable for muscle synergies extraction, each movement was time-normalized to 151 samples. As a result, a matrix of size [(*ns* · *dr* · *r*) × nm] was obtained, where *ns* is the number of samples per movement (151), *dr* is the number of movement planes (3), *r* represents the number of repetitions for each movement plane (it was fixed to 6 for all subjects and both experimental sessions; no trials were excluded from the analysis), and *nm* is the number of muscles (20). Afterwards, this matrix was used to extract spatial muscle synergies applying NMF algorithm (Lee and Seung 1999). The NMF decomposes the EMG data matrix into the product of two matrices, the first one containing time-invariant synergies (Wi) and the second one representing time-varying activation coefficients for each synergy (Ci): $\:EMG\:=\:\sum\:_{i=1}^{N}{C}_{i}\left(t\right){W}_{i}$, where N is the total number of extracted synergies. NMF was performed using MATLAB’s built-in nnmf function with default settings, including random initialization and the alternative least squares minimizing the Euclidean distance between the original and reconstructed EMG metrices. For each selected number of synergies (ranging from 1 to 20), the algorithm was run 500 times to reduce the risk of convergence to local minima, and the solution yielding the highest reconstruction quality was retained. Reconstruction quality was quantified using the coefficient of determination (R^2^), computed between the original and reconstructed EMG matrices. R^2^ was preferred over variance accounted for (VAF) value as it provides a more conservative estimate of reconstruction accuracy. Indeed, as is well established in the literature, R^2^ values are generally lower than VAF values when computed for the same number of synergies (Zhao et al. 2025); therefore, the mean R^2^ value obtained in the present study can be considered acceptable. For this study, a fixed number of four synergies was selected for all subjects, limbs and sessions to ensure a consistent dimensionality of the model across conditions and to avoid solutions with excessively sparse synergies (Severini and Zych 2020; Zhao et al. 2021). This choice provided comparable R^2^ values across conditions and a compromise between reconstruction quality and model simplicity, enabling reliable inter-session comparison. However, the aim of the study was to assess inter-session similarity of synergy vectors rather than dimensionality, therefore the number of synergies was not treated as an outcome variable.

### Inter-session similarity

After extracting the spatial muscle synergies for each subject in both experimental sessions, synergy weights obtained in the two sessions were compared to assess their similarity using cosine similarity. The inter-session similarity was evaluated separately for movements performed with the dominant (right) and non-dominant (left) upper limbs.

Cosine similarity was used as a measure of synergy similarity, where 1 represents synergies with the same spatial composition and 0 is achieved in case of totally orthogonal synergies (Zhao et al. 2021). For each subject, the synergies obtained in the first session were compared to those from the second session. To identify the optimal matching between synergies across sessions, the pairs were selected based on the highest cosine similarity values. Once the best correspondence between synergies was established, the average similarity across the matched synergies was computed, providing a subject-level measure of inter-session consistency. Finally, these subject-level averages were pooled to compute the overall mean similarity across all participants, separately for the dominant and non-dominant arm. This allowed for the assessment of whether synergy consistency across sessions differed between limbs. Moreover, to provide a reference and determine whether the observed similarity between sessions was meaningful, a random similarity analysis was performed on the dataset of extracted synergies. The random analysis consisted of computing the similarity between randomly paired synergies obtained from the two sessions for each subject individually. The resulting values were then averaged across all subjects for both the dominant and non-dominant arm, serving as baseline references to assess whether the observed inter-session similarity exceeded chance levels, thus indicating non-random repeatability of the extracted synergies.

Successively, after comparing the spatial components of muscle synergies between the two sessions, the similarity of the temporal activation coefficients was also evaluated. For each subject and each session, the temporal coefficients of synergies were grouped according to movement plane (adduction, sagittal and scapular) and averaged across the six repetitions performed within each plane, resulting in one mean temporal profile per plane. The three mean temporal profiles were then concatenated into a single matrix, representing the overall mean temporal activation pattern of the synergies across the three movement planes. The pairing between synergies from the two sessions for the comparison of temporal profiles was defined based on the pairing previously obtained for the spatial components. Accordingly, for each synergy, the corresponding mean temporal profiles between T0 and T1 were compared using the Pearson correlation coefficient. A moderate-to-high correlation indicates that the synergy preserves a similar and temporally synchronized activation pattern across the two sessions.

### Statistical analysis

After assessing data distribution, non-parametric statistics were used to evaluate inter-session repeatability for both limbs and to determine whether differences between the dominant and non-dominant limbs were statistically significant, based on small sample sizes and the lack of normal data distribution. First, Wilcoxon signed-rank test was used to check that inter-session reliability was not due to chance, by comparing mean similarity values of paired muscle synergies between T0 and T1 for each subject with the mean similarity values of randomly mismatched synergies between T0 and T1.* P*-value less than 0.05 was interpreted as evidence that inter-session repeatability was not attributable to random variability. Subsequently, the Wilcoxon signed-rank test was applied to assess whether the inter-session reliability was significantly different between the dominant and non-dominant arm. In this case, the test compared mean similarity values obtained by averaging paired synergy comparisons for the dominant and non-dominant arm across all subjects. Notably, paired synergies used for this comparison were extracted from two different sessions (T0 and T1), ensuring that the analysis captured inter-session behavior for each limb.* P*-values lower than 0.05 indicated that the two arms exhibited significant differences in performing the same motor tasks. Finally, Spearman’s rank correlation coefficient was used to assess the presence of a monotonic association between the average synergy similarity of the dominant and non-dominant arms across subjects. This analysis aimed to determine whether subjects exhibiting higher synergy similarity in the dominant arm also tended to show higher similarity in the non-dominant arm. Statistical significance was set at* p*-value lower than 0.05.

## Results

### Synergies extraction

In this study, 4 synergies were selected to describe the motor tasks performed by participants. This choice was applied across all sessions and for both arms, as these 4 synergies provided a clear representation of the biomechanical role played by synergies during different movement phases. For descriptive purposes, the four extracted synergies were labeled Upper limb acceleration synergy, Upper limb deceleration synergy, Postural adjustment synergy 1 and Postural adjustment synergy 2, according to their predominant muscle involvement and activation timing. The Upper limb acceleration synergy was mainly characterized by the contribution of shoulder flexors and was activated during the initial phase of arm elevation. The Upper limb deceleration synergy primarily involved muscles acting to decelerate and stop the movement near the final position. Postural adjustment synergies 1 and 2 were characterized by a greater involvement of trunk and lower-limb muscles and showed activation profiles consistent with stabilization demands during arm raising and movement termination. A detailed representation of the spatial and temporal activation components of muscle synergies is contained in the Supplementary Information. Specifically, this section includes a representative example illustrating the spatial synergies and their temporal components for the dominant arm at T0.

**Fig. 3:**
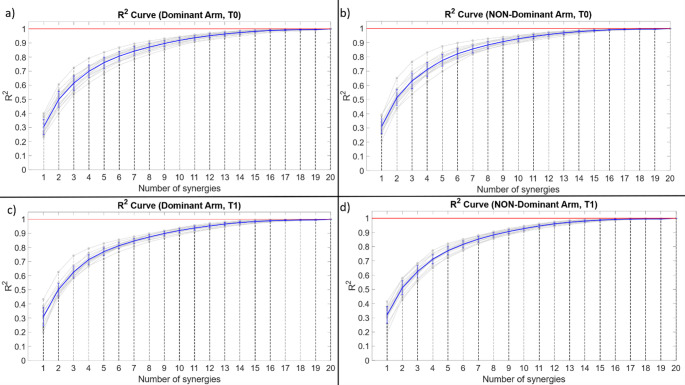
R^2^ curves. The blue line represents the average R^2^ curve across all subjects, the vertical error bars indicate the standard deviation, and the grey curves represent the individual subjects’ R^2^ curves.** a** R^2^ curve of the dominant arm at T0. For four synergies, the mean R^2^ value was 0.70 ± 0.05.** b** R^2^ curve of the non-dominant arm at T0. For four synergies, the mean R^2^ value was 0.71 ± 0.05.** c** R^2^ curve of the dominant arm at T1. For four synergies, the mean R^2^ value was 0.71 ± 0.03.** d** R^2^ curve of the non-dominant arm at T1. For four synergies, the mean R^2^ value was 0.71 ± 0.03

The choice of four synergies was further supported by the R^2^ curves, shown in Fig. 3, which indicate that, with four synergies, the R^2^ values were comparable across conditions and close to 0.70 in all cases, providing a consistent compromise between reconstruction quality and model simplicity and allowing reliable inter-session comparisons.

### Inter-session similarity

**Fig. 4:**
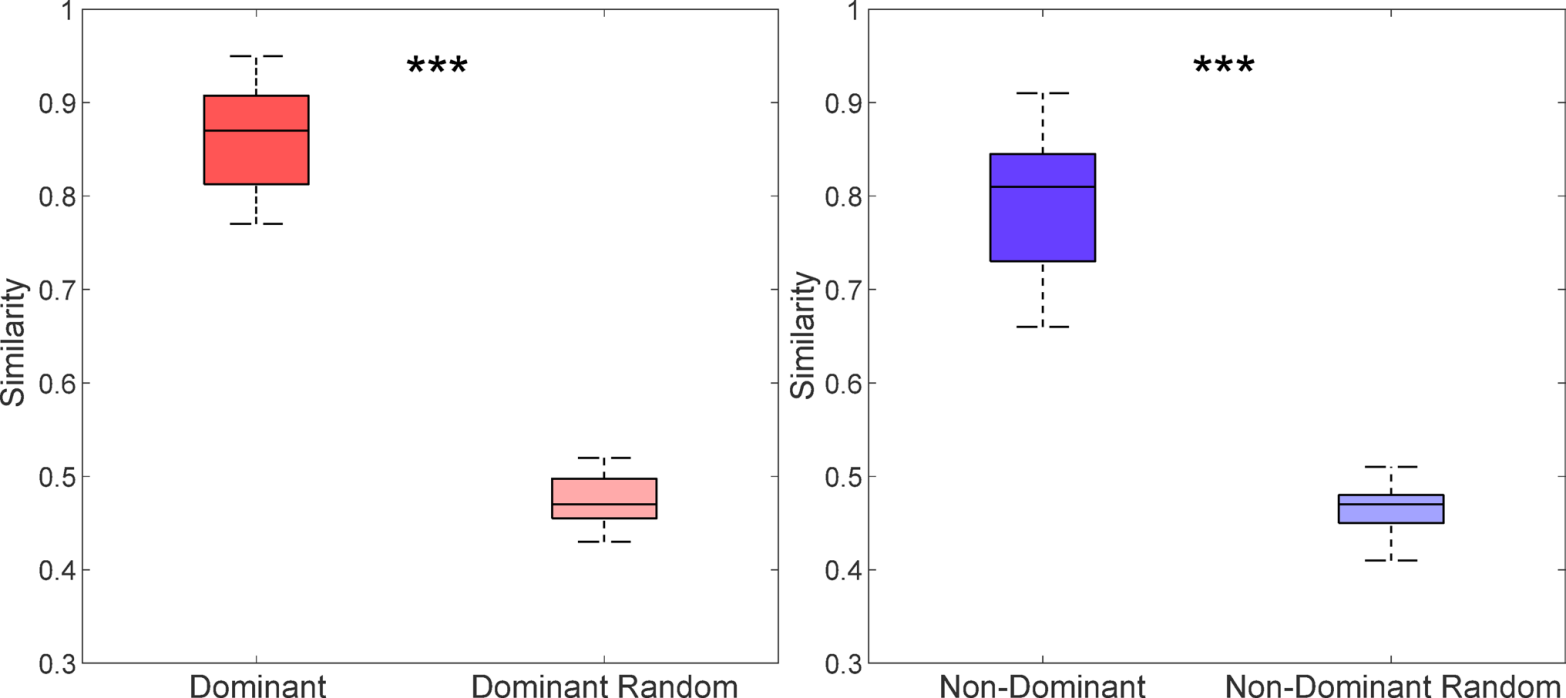
Boxplots show optimal and random similarity values for the dominant and non-dominant arms. The horizontal line inside each box represents the median similarity, while the whiskers indicate the minimum and maximum similarity values observed for each condition. Wilcoxon signed-rank test was performed. *** Statistically significant differences between optimal and random conditions (*p*-value < 0.001)

Wilcoxon signed-rank test revealed a statistically significant inter-session similarity of muscle synergies in both upper limbs (*p*-value < 0.001 in the dominant and non-dominant arms). As shown in Fig. 4, the optimal similarity, obtained by appropriately matching synergies between time points T0 and T1 to maximize correspondence, was significantly higher than random similarity, which was calculated by randomly pairing synergies across sessions. This difference was found in both dominant and non-dominant arms.

**Fig. 5:**
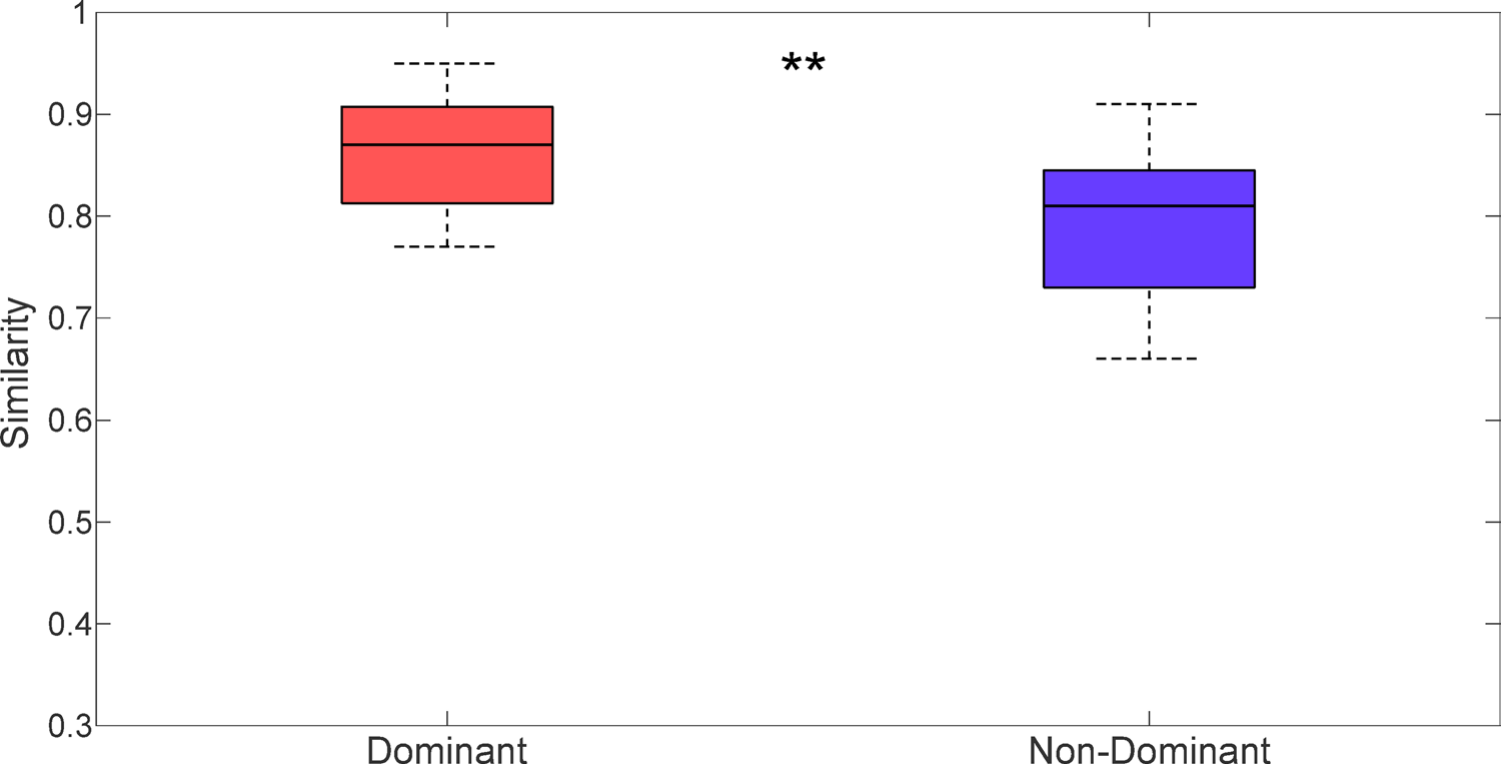
Boxplots show inter-session similarity values for the dominant and non-dominant arms. The horizontal line inside each box represents the median similarity, while the whiskers indicate the minimum and maximum similarity values observed for each condition. Wilcoxon signed-rank test was performed. ** Statistically significant differences between dominant and non-dominant upper limbs (*p*-value < 0.01)

Subsequently, inter-session similarity between the dominant and non-dominant arms was compared, and Wilcoxon signed-rank test revealed a statistically significant difference (*p*-value = 0.002). As highlighted in Fig. 5, the dominant arm showed higher inter-session similarity (0.86) when compared to the non-dominant arm (0.78).

**Fig. 6:**
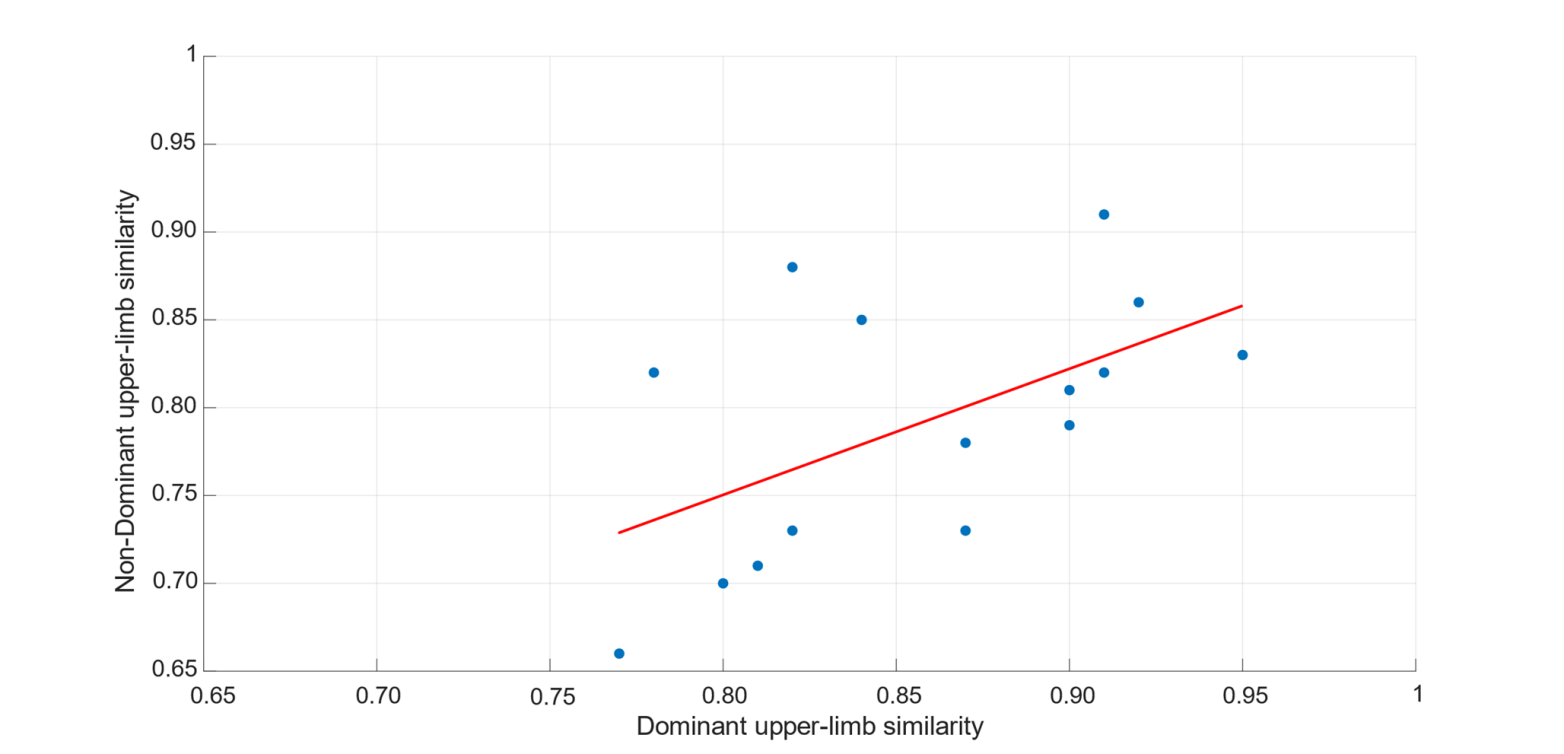
Scatter plot illustrating the monotonic correlation between the average inter-session synergy similarity of the dominant and non-dominant upper-limbs across subjects. Each point corresponds to the average inter-session similarity obtained for a single subject. The red line represents the linear regression fit and is shown for visualization purposes

Finally, Spearman’s rank correlation coefficient was used to assess whether a monotonic correlation existed between the average inter-session synergy similarity of the dominant and that of the non-dominant arm across subjects (*n* = 15). The analysis revealed a statistically significant moderate positive correlation (ρ = 0.59, *p*-value = 0.02) between the inter-session synergy similarity of the dominant and non-dominant arms, as also illustrated in Fig. 6.

### Inter-session similarity and inter-synergies variability: single-subject analysis

When analyzing results of individual subjects, the majority of participants (except S08, S10, S13, and S14) showed higher inter-session median similarity for the dominant arm. Specifically, median similarity values of muscle synergies in the dominant arm ranged from 0.77 to 0.92, with one subject reaching 0.95. In contrast, values of the non-dominant arm were lower, ranging from 0.66 to 0.86, with a single subject reaching 0.91.

**Fig. 7:**
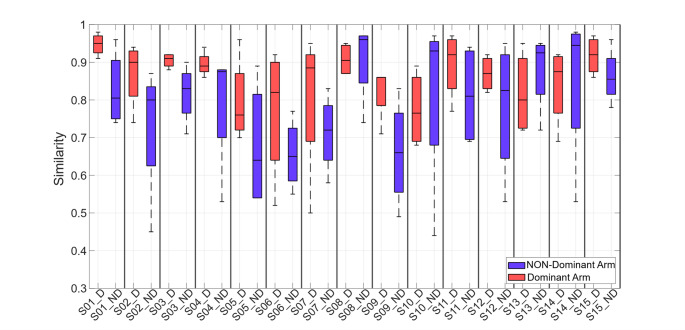
Boxplots show inter-session synergy variability values for each subject, comparing the dominant and non-dominant arms. The horizontal line inside each box represents the median similarity, while the whiskers indicate the minimum and maximum similarity values observed for each subject

To further clarify the difference between arms, Fig. 7 illustrates the repeatability of muscle synergies between sessions for each subject. Each pair of boxplot represents the distribution of similarity values (from 0 to 1) across the four synergies for the dominant and non-dominant arms. Overall, data revealed moderately high inter-session repeatability of synergies with median values exceeding 0.85 in most subjects. Furthermore, for the majority of participants, the dominant arm revealed narrower interquartile ranges (IQR) and higher median values, suggesting greater stability and reduced variability between synergies.

The only exceptions were participants S08, S10, S13 and S14, who exhibited higher median similarity values of muscle synergies in the non-dominant arm. However, as with the other subjects, the IQR was larger for the non-dominant arm, indicating greater inter-synergies variability compared to the dominant arm.

Notably, two subjects (e.g., S06 and S07) exhibited considerable inter-session variability between synergies even in the dominant arm. However, median similarity remained higher compared to the non-dominant side.

### Inter-session similarity and inter-subject variability for each synergy

**Fig. 8:**
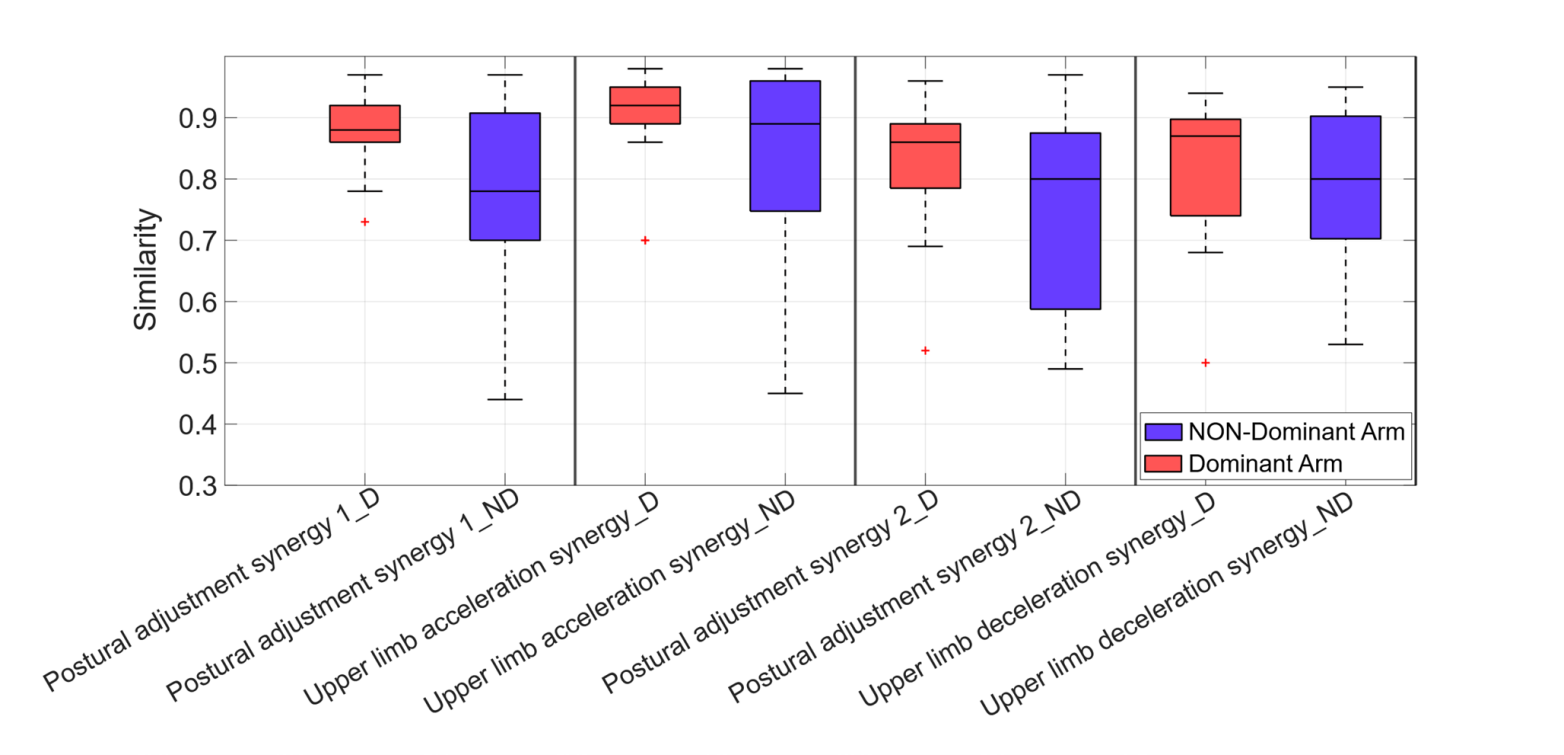
Inter-session similarity of muscle synergies at T0 versus T1. Inter-session repeatability of the four synergies (Upper limb acceleration synergy, Upper limb deceleration synergy, Postural adjustment synergy 1 and Postural adjustment synergy 2) comparing the dominant (red boxplots) and non-dominant (blue boxplots) arms in different subjects. The horizontal line inside each box represents the median similarity, while the whiskers indicate the minimum and maximum similarity values observed for each type of synergy

The inter-session variability of the four synergies (Upper limb acceleration synergy, Upper limb deceleration synergy, Postural adjustment synergy 1 and Postural adjustment synergy 2) was analyzed. Figure 8 illustrates the inter-session consistency of these synergies across T0 and T1 comparing the dominant (D) and non-dominant (ND) arms in different subjects. As shown in the figure, the dominant arm generally showed higher inter-session repeatability across all synergy types. In particular, Upper limb acceleration synergy in the dominant arm revealed the highest median similarity and the narrowest IQR, indicating it is the most stable and consistent synergy across sessions and subjects. Even in the non-dominant arm, Upper limb acceleration synergy showed the highest median similarity, confirming its relatively stable nature. However, the wider IQR indicated greater inter-individual variability compared to the dominant arm. In contrast, Postural adjustment synergy 1 and 2 showed the lowest repeatability, especially for the non-dominant arm, where both the median similarity values were lower and the variability across the subjects was higher. In particular, the Postural adjustment synergy 1 showed the lowest median similarity values, while the Postural adjustment synergy 2 emerged as the most variable across subjects.

The Upper limb deceleration synergy exhibited a comparable variability in both limbs, since the non-dominant arm showed a lower median similarity value, suggesting it was less repeatable over time. Data dispersion was moderate for both arms, suggesting moderate inter-subject variability, which was slightly higher for the non-dominant arm.

### Temporal activation coefficients similarity

Tables 1 and 2 show the Pearson correlation coefficients obtained from the comparison of the temporal activation profiles of corresponding muscle synergies between the two sessions. Table 1 refers to the dominant arm, while Table 2 refers to the non-dominant arm.

**Table 1:** Pearson correlation coefficients obtained by comparing the mean temporal activation profiles of muscle synergies between sessions T0 and T1 for the dominant arm

Subjects	Upper-limb acceleration synergy	Upper-limb deceleration synergy	Postural adjustment synergy 1	Postural adjustment synergy 2
S01	0.79 *(p < 0.001)*	0.95 *(p < 0.001)*	0.68 *(p < 0.001)*	0.60 *(p < 0.001)*
S02	0.92 *(p < 0.001)*	0.82 *(p < 0.001)*	0.82 *(p < 0.001)*	0.74 *(p < 0.001)*
S03	0.91 *(p < 0.001)*	0.92 *(p < 0.001)*	0.85 *(p < 0.001)*	0.81 *(p < 0.001)*
S04	0.68 *(p < 0.001)*	0.78 *(p < 0.001)*	0.37 *(p < 0.001)*	0.65 *(p < 0.001)*
S05	0.71 *(p < 0.001)*	0.70 *(p < 0.001)*	0.49 *(p < 0.001)*	0.49 *(p < 0.001)*
S06	0.91 *(p < 0.001)*	0.41 *(p < 0.001)*	0.64 *(p < 0.001)*	0.51 *(p < 0.001)*
S07	0.85 *(p < 0.001)*	0.59 *(p < 0.001)*	0.43 *(p < 0.001)*	0.50 *(p < 0.001)*
S08	0.94 *(p < 0.001)*	0.91 *(p < 0.001)*	0.89 *(p < 0.001)*	0.45 *(p < 0.001)*
S09	0.63 *(p < 0.001)*	0.76 *(p < 0.001)*	0.70 *(p < 0.001)*	0.74 *(p < 0.001)*
S10	0.60 *(p < 0.001)*	0.46 *(p < 0.001)*	0.52 *(p < 0.001)*	0.45 *(p < 0.001)*
S11	0.87 *(p < 0.001)*	0.57 *(p < 0.001)*	0.91 *(p < 0.001)*	0.61 *(p < 0.001)*
S12	0.92 *(p < 0.001)*	0.67 *(p < 0.001)*	0.78 *(p < 0.001)*	0.68*(p < 0.001)*
S13	0.96 *(p < 0.001)*	0.54 *(p < 0.001)*	0.76 *(p < 0.001)*	0.70 *(p < 0.001)*
S14	0.89 *(p < 0.001)*	0.68 *(p < 0.001)*	0.63 *(p < 0.001)*	0.48 *(p < 0.001)*
S15	0.82 *(p < 0.001)*	0.83*(p < 0.001)*	0.63 *(p < 0.001)*	0.56 *(p < 0.001)*

**Table 2:** Pearson correlation coefficients obtained by comparing the mean temporal activation profiles of muscle synergies between sessions T0 and T1 for the non-dominant arm

Subjects	Upper-limb acceleration synergy	Upper-limb deceleration synergy	Postural adjustment synergy 2	Postural adjustment synergy 1
S01	0.79 *(p < 0.001)*	0.74 *(p < 0.001)*	0.55 *(p < 0.001)*	0.59 *(p < 0.001)*
S02	0.47 *(p < 0.001)*	0.87 *(p < 0.001)*	0.52 *(p < 0.001)*	0.93 *(p < 0.001)*
S03	0.83 *(p < 0.001)*	0.64 *(p < 0.001)*	0.80 *(p < 0.001)*	0.41 *(p < 0.001)*
S04	0.77 *(p < 0.001)*	0.65 *(p < 0.001)*	0.55 *(p < 0.001)*	0.71 *(p < 0.001)*
S05	0.87 *(p < 0.001)*	0.86 *(p < 0.001)*	0.09 *(p = 0.055)*	0.41 *(p < 0.001)*
S06	0.78 *(p < 0.001)*	0.54 *(p < 0.001)*	0.59 *(p < 0.001)*	0.74 *(p < 0.001)*
S07	0.69 *(p < 0.001)*	0.53 *(p < 0.001)*	0.77 *(p < 0.001)*	0.41 *(p < 0.001)*
S08	0.97 *(p < 0.001)*	0.94 *(p < 0.001)*	0.60 *(p < 0.001)*	0.80 *(p < 0.001)*
S09	0.33 *(p < 0.001)*	0.74 *(p < 0.001)*	0.71 *(p < 0.001)*	0.54 *(p < 0.001)*
S10	0.94 *(p < 0.001)*	0.91 *(p < 0.001)*	− 0.09 *(p = 0.0446)*	0.86 *(p < 0.001)*
S11	0.90 *(p < 0.001)*	0.67 *(p < 0.001)*	0.36 *(p < 0.001)*	0.29 *(p < 0.001)*
S12	0.30 *(p < 0.001)*	0.36 *(p < 0.001)*	0.74 *(p < 0.001)*	0.74 *(p < 0.001)*
S13	0.87 *(p < 0.001)*	0.81 *(p < 0.001)*	0.64 *(p < 0.001)*	0.68 *(p < 0.001)*
S14	0.93 *(p < 0.001)*	0.82 *(p < 0.001)*	0.31 *(p < 0.001)*	0.90 *(p < 0.001)*
S15	0.85 *(p < 0.001)*	0.74 *(p < 0.001)*	0.16 *(p < 0.001)*	0.62 *(p < 0.001)*

Referring to Tables 1 and 2, it can be observed that the temporal coefficients showed a variable linear correlation between T0 and T1, depending on the type of synergy considered and on whether the dominant or non-dominant upper limb was analyzed. Specifically, results showed that all temporal activation profiles of the synergies for the dominant arm exhibited a significant linear correlation between T0 and T1 (Table 1). The upper-limb acceleration synergy exhibited the highest and least variable correlation values across subjects, whereas postural adjustment synergy 2 showed the lowest Pearson coefficients, with only a few subjects displaying moderately high linear correlation between the two sessions for this synergy. Instead, postural adjustment synergy 1 and the upper-limb deceleration synergy showed greater inter-subject variability, with some subjects presenting moderately good linear correlation between sessions and others showing lower correlation values, indicating differences in the temporal activation profiles.

A similar result can be observed in Table 2 for the temporal activation coefficients of muscle synergies observed for the non-dominant arm. However, in this case, some subjects exhibited lower correlation values even for the agonist synergy responsible for arm elevation (upper-limb acceleration synergy), and correlation values further decreased in some subjects for the other synergies. Notably, one subject showed a non-significant correlation and another exhibited a very low correlation when comparing the temporal activation profiles of postural adjustment synergy 2.

## Discussion

### Summary of the results

The aim of this study was to investigate the inter-session variability of muscle synergies in healthy subjects who performed upper limb elevation movements in 2 different days with the dominant and non-dominant upper limbs. For each session, four synergies were selected based on the R^2^ value, which exceeded 0.70 in all cases.

To evaluate the variability of muscle synergies across sessions, the four muscle synergies identified at T0 were compared with those obtained at T1. The results showed that muscle synergies exhibited moderately high repeatability: the optimal similarity between synergies (0.86 for the dominant limb and 0.78 for the non-dominant limb) was significantly higher than random similarity (0.48 for the dominant limb and 0.47 for the non-dominant limb), indicating a non-random pattern of repeatability between sessions. Inter-session similarity was compared between the two arms, showing greater repeatability for the dominant arm compared to the non-dominant arm. Moreover, a significant moderate correlation was observed between inter-session similarity in the dominant and non-dominant upper-limb (ρ = 0.59), suggesting that subjects with higher inter-session similarity in the dominant arm also tended to show higher similarity in the non-dominant arm. This indicated a partial conservation of synergy organization across limbs, reflecting a shared or consistent motor control strategy. Finally, the repeatability of each individual synergy type was analyzed, based on the biomechanical role of each synergy during the tasks. In general, all synergy types were more repeatable in the dominant arm compared to the non-dominant arm. In particular, the Upper limb acceleration synergy at the level of the dominant arm, resulted as the most repeatable synergy across sessions (about 0.90), probably being the synergy related to the agonist component of the upper limb elevation task. In fact, the Upper limb acceleration synergy also had the highest inter-session similarity in the non-dominant arm (about 0.85). In contrast, the Postural adjustment synergies showed lower repeatability, especially in the non-dominant arm, where the median similarity was lower, and the variability across the subjects resulted to be higher. This effect was most pronounced for the Postural adjustment synergy 1, which proved to be the synergy with the lowest stability between the two sessions.

Moreover, for each synergy, the temporal activation coefficients similarity was also evaluated between the two sessions, showing variable repeatability depending on the type of synergy for both arms. In particular, the upper limb acceleration synergy for both the dominant and non-dominant arm showed a moderate-to-high linear correlation, whereas greater inter-subject variability and lower correlations were observed for postural synergies, especially for postural adjustment synergy 2. This effect was more evident in the non-dominant arm, where some subjects exhibited weak or non-significant correlations.

### Synergies are consistently structured between sessions, but show non-negligible differences

Four muscle synergies were extracted at T0 and T1. For descriptive purposes, these synergies were labeled to distinguish those primarily responsible for arm movement from those mainly involved in body stabilization during the tasks (Upper limb acceleration synergy, Upper limb deceleration synergy, Postural adjustment synergy 1 and Postural adjustment synergy 2). The main focus of this study is on assessing the inter-session similarity of synergy weighting coefficients, rather than providing a detailed biomechanical characterization of the synergies, which will be addressed in a separate investigation and is beyond the scope of the present work. Thus, this study is exclusively concerned with evaluating the inter-session repeatability of muscle synergies. Within this context, the results indicate that muscle synergies exhibit a consistent non-random structure across sessions. However, this evidence alone does not allow for a direct quantitative interpretation of their level of repeatability, which instead requires comparison with findings reported in the existing literature. By appropriately pairing the synergies across sessions, their similarity was found to be significantly higher than similarity detected with randomly paired synergies. This result demonstrated that synergies exhibited a statistically significant similarity and non-random repeatability across sessions, as expected and proved in previous studies (D’Avella and Bizzi 2005). However, a closer inspection reveals that, although biomechanical roles of synergies appeared to be unchanged between sessions, they did not always exhibit identical muscle weights. Specifically, the relative contribution of individual muscles within each synergy may vary, as suggested by similarity values. Indeed, inter-session similarity ranged from 0.77 to 0.92 in the dominant arm, while similarity ranged from 0.66 to 0.85 in the non-dominant arm. Such differences may be explained through many factors. First, slight inaccuracies in sensor positioning or during gesture execution might have occurred, although these effects were kept to a minimum in the protocol and through particular care of the experimenters. Second, a physiological variability in terms of movement pattern might have occurred between sessions due to the inherent characteristics of redundancy of the musculoskeletal system, which allows for the performance of the same movement through different muscle recruitment strategies (D’Avella and Bizzi 2005; Latash et al. 2002). This last effect may be especially evident in Postural adjustment synergies, as the recruitment of muscles included in these synergies may be influenced by the subjects’ initial posture in addition to the mechanical demand. Similar findings were reported by Pale and co-workers who observed inter-session similarity values between 0.70 and 0.84 when analyzing hand grasps and forearm muscle activations (Pale et al. 2020). Therefore, while some variability in synergy composition was found, the overall similarity between sessions can still be considered moderately high for both arms.

Importantly, this variability should be interpreted in the context of neuromuscular control of upper limb movements. In fact, synergies described in lower limbs during gait or at the level of trunk muscles during postural control tend to be more stable over time, while upper limb synergies appear to be more variable (Jobson et al. 2013; Saito et al. 2023). This behavior may occur as a consequence of high-level movement control required during upper limb tasks. Upper limb motion requires different control from that needed for stereotypical movement patterns occurring during some lower limb tasks, such as walking. In fact, a certain degree of variability is physiological during upper limb motion, where movement flexibility represents a compromise between movement accuracy and minimization of metabolic cost. In addition, continuous adaptation of upper limb motor patterns to environmental conditions is often required. Again, the neuromotor system may recruit different muscle combinations depending on slight variations in intrinsic (e.g., fatigue, motivation, attention) or extrinsic (e.g., posture, task constraints, feedback) conditions, which may change between sessions (Cheung et al. 2012). As such, similarity values reported in this study were not only statistically significant, but also meaningful in describing the similarity of upper limb muscle synergies. Therefore, it is worth underlining that observed differences should not be interpreted as a limitation, but as a reflection of adaptive nature of motor control in humans. These factors deserve to be considered when designing longitudinal studies aimed at assessing motor improvements, since subtle changes in biomechanical context or subjects’ clinical features might influence synergy composition over time.

### Quantifying inter-session variability for longitudinal assessments

The quantification of synergies variability is essential to understand the actual changes in motor performance other than physiological variability characterizing human movements. In our study, muscle synergies in healthy subjects performing multi-planar movements repeated across sessions were assessed. Moderately high repeatability of synergies was expected, since no motor training or changes in clinical conditions of subjects occurred between sessions. However, some differences were found, suggesting the presence of some factors able to affect inter-session repeatability of synergies in healthy subjects (Zhao et al. 2021). The relevant consequence, already highlighted in previous studies (Pale et al. 2020; Saito et al. 2023), is that changes in motor control found in multi-session trials should not necessarily be related to neural plasticity or motor recovery alone. In fact, such variations may arise from a range of factors, including natural inter- and intra-subject variability, intrinsic factors that fluctuate in time (e.g., fatigue, stress, mood), and extrinsic influences such as differences in electrode placement between sessions (Pale et al. 2020). It is therefore critical to distinguish these sources of variability from those changes that reflect actual improvements in neuromuscular control.

In this study, the average similarity of synergies between sessions was 0.86 for the dominant arm and 0.78 for the non-dominant arm. These values revealed a certain variability, despite the absence of any intervention intended to alter motor strategies. As such, observed differences may be attributed to physiological variability of human movements rather than true changes in motor control (Torres-Oviedo and Ting 2010). This variability found in healthy subjects can therefore be considered a sort of “baseline” variability, which should be considered in longitudinal studies and taken into account in interpreting motor performance changes in clinical contexts. Consequently, longitudinal investigations aimed at evaluating motor improvements should first establish the variability threshold in a healthy subject in order to determine whether observed changes reflect true neuroplastic adaptations or variability introduced by other factors.

### High dynamics, limb dominancy and biomechanical role of synergies impact on variability

A key aspect that may have influenced the level of similarity in muscle synergies between sessions is the type of movement and the selection of assessed muscles. In particular, our analysis revealed notable differences in the repeatability of synergies based on their biomechanical role, especially between synergies involved in generating the agonist movement component (Upper limb acceleration synergy, Upper limb deceleration synergy) and those responsible for postural control in terms of anticipatory postural adjustments and joint stabilization (Postural adjustment synergy 1 and Postural adjustment synergy 2).

Upper limb acceleration synergies in both dominant and non-dominant arms exhibited higher repeatability across sessions. In contrast, Postural Adjustment synergies showed lower similarity, indicating greater variability. These findings suggested that synergy related to agonist movement components (Upper limb acceleration synergies) are more stable between sessions, while those associated with postural control (Postural adjustment synergies) are more susceptible to inter-session variability. This distinction was also evident when considering the correlation between the temporal activation profiles of muscle synergies across T0 and T1. Upper-limb acceleration synergies in both dominant and non-dominant arms exhibited a moderate-to-high linear correlation across sections, indicating temporally synchronized activation of the muscle synergies during the performed movements. In contrast, Postural adjustment synergy 2 showed lower correlation, reflecting greater variability in the timing activation of this synergy which stabilized the body prior to the moment of stopping the arm in the final position. Additional differences in the temporal correlations may be explained by slight shifts in the activation timing of individual synergies, depending on how the movement was performed by each subject during the two sessions, resulting in major variations in the motor pattern.

Moreover, the dominant arm consistently demonstrated higher inter-session similarity across all synergy types. Notably, Upper limb acceleration synergy at the level of the dominant arm emerged as the most repeatable and least variable synergy across subjects. This result may indicate that motor control strategies for performing motor tasks with the dominant arm are not only more stable but also less influenced by subject-specific or environmental factors. One possible explanation is greater motor dexterity typically associated with limb dominance (Bagesteiro and Sainburg 2002). These differences between the dominant and non-dominant arms may be further amplified by the specific characteristics of the chosen task, which involved a highly dynamic movement requiring precise, explosive, execution, and abrupt stabilization. Achieving a similar level of control with the non-dominant arm may be more challenging for most individuals, causing an increase in variability of synergies in that limb (Bagesteiro and Sainburg 2002).

In conclusion, the similarity of muscle synergies seems to be influenced by both the nature of the motor task and the experimental design, including muscles and limbs selected for analysis. These factors should be carefully considered when interpreting results and assessing the repeatability of muscle synergies in longitudinal studies.

### Biases and limitations

Some limitations deserve to be highlighted. First, the EMG filtering pipeline may have introduced sources of variability that affected the repeatability of muscle synergies across sessions. Currently, there is no universally accepted pipeline for muscle synergy analysis, and different preprocessing choices, including filtering, time normalization, and dimensionality reduction, can significantly affect the outcomes (Kieliba et al. 2018; Steele et al. 2013). In particular, the normalization procedures applied to EMG signals can introduce artifacts in muscle activation profiles, potentially altering the extracted synergies and increasing variability between sessions.

Additionally, the number of subjects included in this study was relatively small. Future research should determine whether the repeatability of synergies improves with a larger cohort or whether the variability patterns observed here are consistently maintained. Another limitation concerns the age range of the participants, which was restricted to young adults, limiting the generalizability of the current findings. Age-related changes in neuromuscular control may affect the organization and variability of muscle synergies (Delis et al. 2018; Monaco et al. 2010). Furthermore, gender-related differences in motor control and muscle activation patterns have been suggested in some studies (Hunter 2016; Schmitz et al. 2007) and this aspect was not specifically investigated in our analysis. Future research should assess the impact of gender on muscle synergies variability through gender-based analysis. Moreover, a detailed kinematic characterization of the movement was lacking. All subjects received identical instructions in both sessions, and movement execution was visually supervised to ensure task consistency. However, this approach may not guarantee precise standardization of the movement, meaning that it may not always be executed in exactly the same way, which could slightly contribute to minor variations in the extracted synergies. Therefore, future studies could benefit from incorporating quantitative kinematic assessments, which may help to better standardize the movement and improve the repeatability of motor control during task execution. Lastly, the current analysis was limited to a single motor task, even if multi-planar. Including a broader range of motor gestures would provide a more comprehensive representation of neuromuscular control and allow for a better understanding of how synergy repeatability may vary across tasks. Such expansion would enhance the applicability of the findings in both research and clinical settings.

## Conclusion

Muscle synergies extracted during multi-planar upper limb elevation tasks showed a moderate-to-high degree of inter-session similarity in healthy subjects, but non-negligible variability was also observed. This variability cannot be attributed to a single factor, but rather to multiple sources related to both motor control and experimental conditions. First, variability depended on the biomechanical role of the synergies: synergies mainly associated with arm acceleration and deceleration were more repeatable across sessions, whereas postural adjustment synergies showed greater variability, particularly in the non-dominant limb. Second, limb dominance influenced inter-session consistency, with higher repeatability observed in the dominant arm compared with the non-dominant arm, highlighting the role of motor proficiency and task familiarity. Third, the characteristics of the motor task and the experimental design, including the use of multi-planar, highly dynamic movements and the involvement of both upper limb and postural muscles, contributed to the observed level of variability. Finally, methodological factors related to data acquisition and processing may also affect synergies estimation and should be carefully considered in longitudinal studies. Overall, these findings emphasize that inter-session variability of muscle synergies reflects a combination of physiological, biomechanical and methodological factors. Quantifying this variability in healthy subjects provides a necessary reference for interpreting longitudinal changes in motor control and for distinguishing true functional adaptations from natural or methodological fluctuations.

## Supplementary Information

Below is the link to the electronic supplementary material.

Supplementary Material 1

## Data Availability

The data presented in this study are available on request from the corresponding authors and pending the ethical committee approval.
