# Supplementary material for: Inter-session variability of muscle synergies during upper limb elevation movements in healthy subjects
Source: Exp Brain Res. 2026 Apr 19;244(5):95. doi: 10.1007/s00221-026-07297-8 (PMC13092520; doi:10.1007/s00221-026-07297-8)
Supplement: Supplementary file 1 — Supplementary Material 1 [file 221_2026_7297_MOESM1_ESM.docx]

**Supplementary Information**

Title: Inter-session variability of muscle synergies during upper limb elevation movements in healthy subjects

Journal: Experimental Brain Research

Authors: Valentina Lanzani^1^, Francesco Scandelli^2^, Federico Temporiti^2,3^, Francesca Cappelletti^4^, Luca Canova^4^, Paola Adamo^2^, Roberto Gatti^2,3^, Alessandro Scano^1^

^1^ Institute of Intelligent Industrial Systems and Technologies for Advanced Manufacturing (STIIMA), Advanced Methods for Biomedical Signal and Image Processing Laboratory, Italian Council of National Research (CNR), Milan, Italy

^2^ Physiotherapy Unit, IRCCS Humanitas Research Hospital, Rozzano, Milan, Italy

^3^ Department of Biomedical Sciences, Humanitas University, via Rita Levi Montalcini 4, 20072 Pieve Emanuele, Milan, Italy

^4^ Humanitas University, via Rita Levi Montalcini 4, 20072 Pieve Emanuele, Milan, Italy

**Results**

*Synergies extraction*

A representative example illustrates the spatial synergies (Figure 1) and their temporal components (Figure 2) for the dominant arm at T0.


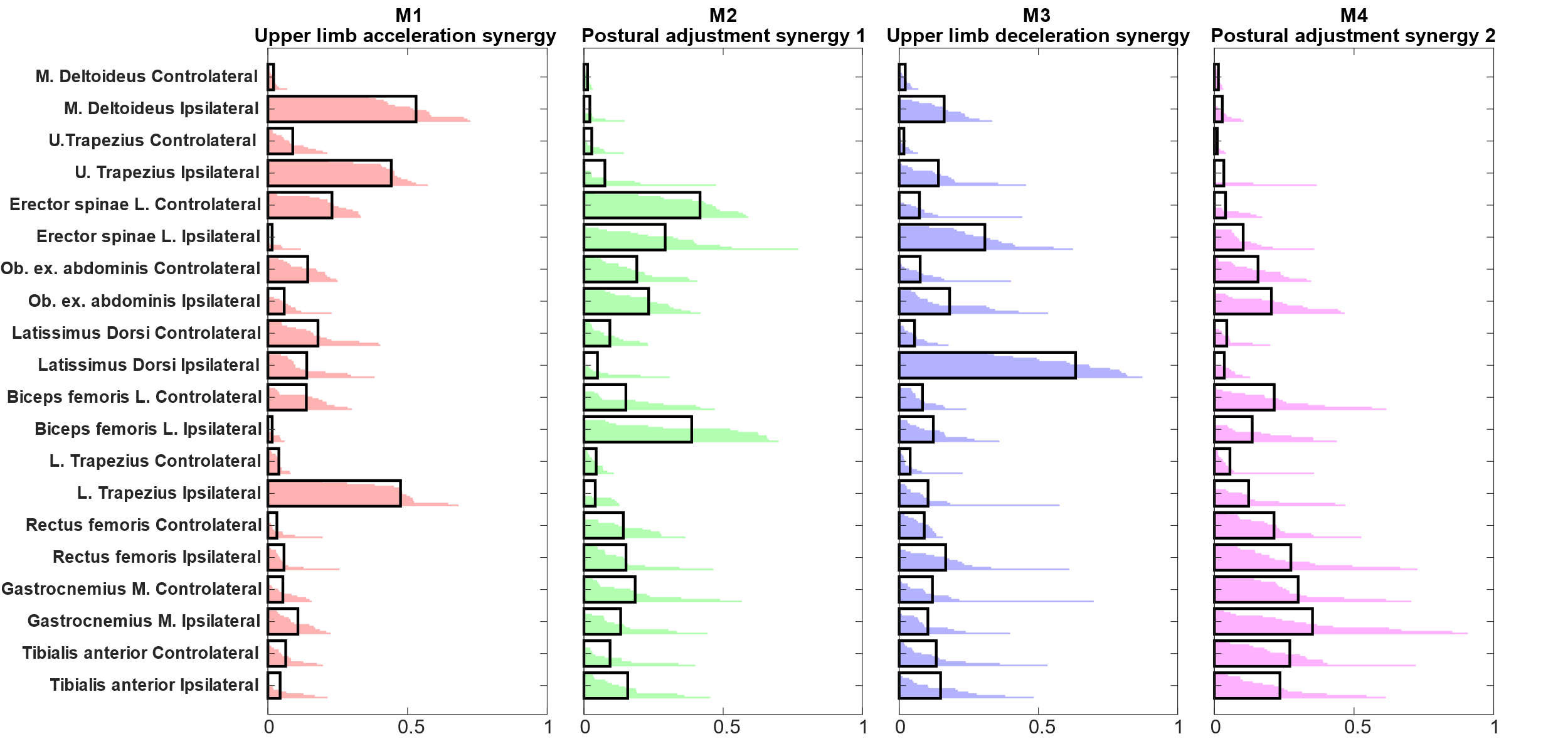


**Fig. 1** Group-averaged spatial muscle synergies of the dominant arm at T0, describing the contribution of each muscle to the coordination pattern. Black bars represent the mean normalized muscle weighting coefficients across subjects, whereas the colored traces correspond to individual subjects’ spatial synergies. For descriptive purposes, synergies are labeled according to their predominant muscle involvement: upper-limb acceleration, upper-limb deceleration, postural adjustment synergy 1 and postural adjustment synergy 2. The muscles considered bilaterally are: Middle Deltoideus (M. Deltoideus), Upper Trapezius (U. Trapezius), Erector spinae Longissimus (Erector spinae L.), Obliquus Externus Abdominis (Ob. ex. abdominis), Latissimus Dorsi (Latissimus Dorsi), Biceps Femoris Long Head (Biceps femoris L.), Rectus Femoris (Rectus femoris), Gastrocnemius medialis (Gastrocnemius M.), Tibialis Anterior (Tibialis anterior).

**
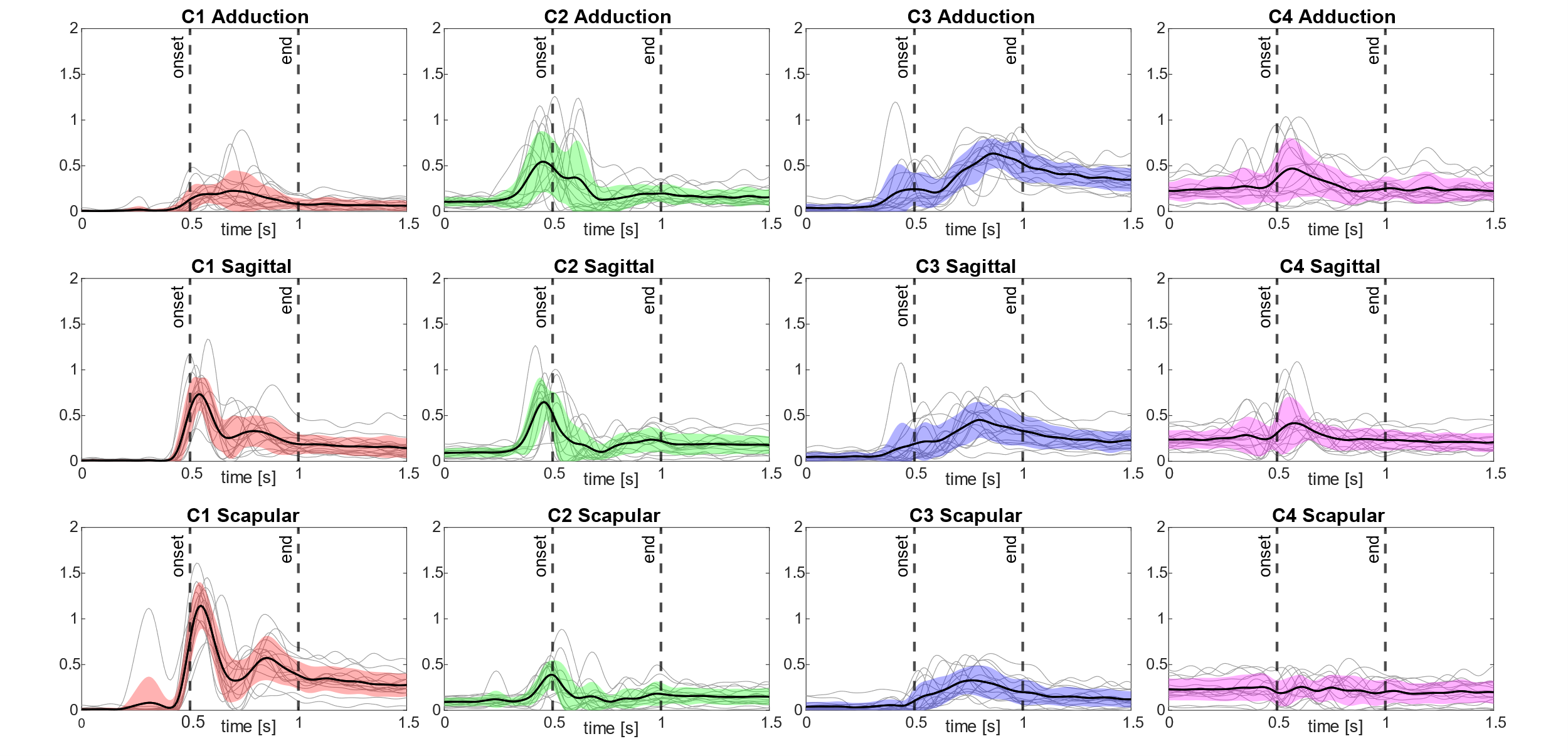
**

**Fig. 2** Group-averaged temporal coefficients of the spatial muscle synergies of the dominant arm at T0, indicating the phase of the movement in which each spatial synergy is activated. The black line represents the mean temporal profile across all subjects, the colored area indicates the standard deviation, and the gray lines represent individual subjects’ temporal profile.
